# Losartan, toceranib, and carboplatin combination therapy for progressive pulmonary metastasis in canine osteosarcoma: a case report

**DOI:** 10.1186/s13620-026-00337-3

**Published:** 2026-03-02

**Authors:** Ji-Hoon Kwak, Mingyu Jung, Soo-Nyun Choi, Kyu-Shik Jeong

**Affiliations:** 1 FM Animal Medical Center, Gimpo City, 10080 Republic of Korea; 2 College of Veterinary Medicine, Kyungpook National University, Daegu, 41566 Republic of Korea; 3 Department of Pet Industry, College of Adventure, Daegu Haany University, 1 Haanydaero, Gyeongsan City, Gyeongsangbuk-Do 38610 Republic of Korea; 4 Stellamed Co., Ltd, Daegu, 41504 Republic of Korea

**Keywords:** Canine Osteosarcoma, Chemotherapy, Losartan, Toceranib, Combination Therapy, Pulmonary Metastasis, Tumor Microenvironment, TME

## Abstract

Osteosarcoma (OSA) is the most common and aggressive primary bone tumor in dogs. OSA frequently metastasizes to the lungs and has a poor prognosis despite current standard treatment. This case report described the clinical course of a 3-year-old castrated male Jindo dog diagnosed with right tibial OSA and a pre-existing pulmonary metastasis. Initially, the patient received adjuvant chemotherapy and maintained a stable disease state for approximately five months (21 weeks from initial diagnosis) until radiographic and computed tomography (CT) examinations revealed progressive pulmonary metastasis.

In response to this progression, the treatment was shifted to a triple-combination regimen of carboplatin, high-dose losartan (angiotensin II type 1 receptor blocker [ARB], 10 mg/kg PO BID), and toceranib (2.75 mg/kg PO EOD). Nine weeks (30 weeks from diagnosis) after initiating this new regimen, a comprehensive CT re-evaluation demonstrated a significant positive response, marked by the radiographic disappearance of pulmonary nodules and complete resolution of the pleural effusion. This substantial improvement indicated a potential clinical benefit of the triple-combination regimen, which may suggest a synergistic effect when used alongside concurrent carboplatin cycles, in addition to losartan’s proposed capacity to remodel the tumor microenvironment (TME) and enhance toceranib efficacy. However, following the conclusion of the carboplatin cycles at week 30, the patient’s condition ultimately deteriorated despite the continuation of losartan and toceranib combination therapy, confirming extensive disease progression by week 40. A subsequent attempt with doxorubicin as a third-line agent resulted in severe adverse effects, including bone marrow suppression and life-threatening neutropenia, leading to the patient’s death shortly thereafter (43 weeks from diagnosis).

This case presents a notable clinical observation of a favorable short-term response of high-dose losartan in combination with toceranib for progressive pulmonary metastatic OSA, potentially through favorable TME modulation. It also highlighted the inherent virulence of canine OSA and the challenges in achieving sustained long-term control. The development of innovative multimodal therapeutic approaches for the management of canine OSA is critically important.

## Introduction

Osteosarcoma (OSA) is the most prevalent primary malignant bone tumor in dogs, accounting for over 85% of all tumors [1, 2]. It commonly occurs in the appendicular skeleton of older dogs, especially large and giant breeds [1, 2]. OSA is characterized by rapid growth and a high propensity for metastasis to other organs, particularly the lungs, often at the time of diagnosis [1–3].

Patients typically present with progressive lameness, pain, and localized edema at the tumor site. The diagnosis of canine OSA is typically made based on clinical findings, imaging, including radiography and computed tomography (CT), and confirmatory cytology or histopathology. The current standard of care for appendicular OSA is surgical amputation of the affected limb, followed by adjuvant chemotherapy [1–4].

Despite interventions, the prognosis of OSA remains poor, owing to its inherent virulence and high metastatic potential in the lungs. While prognosis can vary based on factors such as tumor size, presence of macroscopic metastasis, and the dog’s overall health at diagnosis, the average survival time is approximately 12 months with combined surgery and chemotherapy [1–3]. However, the prognosis for dogs presenting with macroscopic pulmonary metastasis (Stage III disease) is significantly worse, with reported median survival times typically ranging from 2 to 3 months (approximately 59 to 76 days) despite treatment [5, 6] As the long-term outcomes of current standard therapies have remained stagnant for decades, innovative treatments are critically needed to improve the quality of life of affected patients [4]. In advanced or metastatic cancers, where a cure is unlikely, the primary objective shifts from complete remission to slowing disease progression and maximizing the patient’s survival time and quality of life, making strategies to prevent recurrence and metastasis crucial.

Recently, Regan et al. published a landmark study on the combined administration of losartan (a well-known ARB antihypertensive drug used in humans and dogs) and toceranib in dogs with pulmonary osteosarcoma metastasis. Their research reported that high-dose losartan (10 mg/kg BID) combined with toceranib (2.75 mg/kg EOD) achieved a 40% overall response rate (ORR, partial and complete responses) and a 50% clinical benefit rate (CBR, including stable disease), resulting in prolonged survival compared with toceranib monotherapy [7]. Regan et al. suggested that losartan possesses anticancer properties by inhibiting cancer metastasis. This effect is thought to occur through the inhibition of the C-C motif chemokine ligand 2 (CCL2)-C-C chemokine receptor type 2 (CCR2) monocyte recruitment within the tumor microenvironment (TME) [7]. Toceranib, a multi-targeted tyrosine kinase inhibitor (TKI), has been approved for the treatment of canine mast cell tumors owing to its antitumor effects via c-Kit inhibition. It also exhibits significant anti-angiogenic effects by inhibiting platelet-derived growth factor receptor (PDGFR) and vascular endothelial growth factor receptor (VEGFR), making it a widely used targeted anticancer drug in veterinary medicine [8].

The significance of this finding extends beyond veterinary medicine, as canine osteosarcoma is considered an excellent spontaneous model for the human disease, driving new therapeutic strategies through drug repurposing [9].

This case provided a foundation for our therapeutic approach. Here, we evaluated the clinical response to a triple-combination regimen (losartan, toceranib, and carboplatin) in a Jindo dog with progressive pulmonary metastatic osteosarcoma. Our goal was to explore the feasibility of this novel regimen in mitigating progressive pulmonary metastasis and potentially improving patient outcomes.

### Case description

**Fig. 1:**
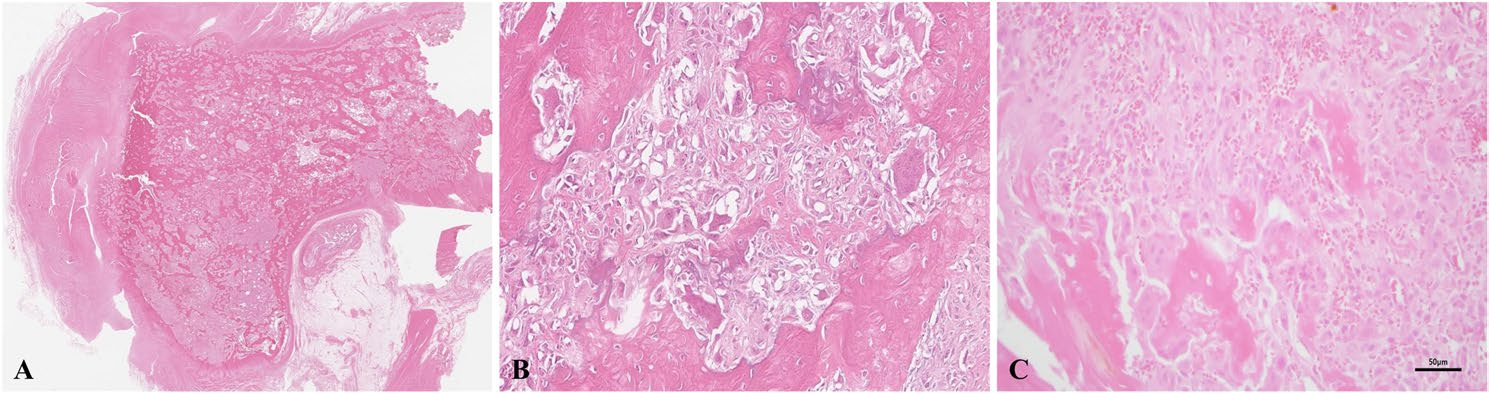
Histological hallmark of osteosarcoma (hematoxylin and eosin staining). (**A**) Low magnification image showing the overall architecture of the osteosarcoma lesion. The tumor extensively infiltrates the bone tissue, characterized by the destruction of normal bone structures. (**B**) The intermediate magnification image revealing the characteristic histology of osteosarcoma, including malignant osteoblastic features. Irregularly-shaped eosinophilic osteoid is seen, surrounded by highly pleomorphic neoplastic osteoblasts. Multinucleated giant cells are also observed. (**C**) The high magnification image highlighting the cytological atypia of the neoplastic cells. Malignant osteoblasts with atypical nuclei and coarse chromatin are actively producing osteoid. The presence of hemorrhage within the tumor stroma is also noted

A 3-year-old castrated male Jindo was diagnosed histopathologically with OSA of the right tibia on April 30, 2024 (week 0) (Fig. 1). Two weeks later, following the right hind limb amputation, the patient was referred to the FM Animal Medical Center on May 24, 2024, for adjuvant chemotherapy. Presurgical staging, which included thoracic computed tomography (CT) (Fig. 4A) and thoracic radiography, revealed pulmonary metastasis, but no spread to the regional lymph nodes or other organs was observed.

**Fig. 2:**
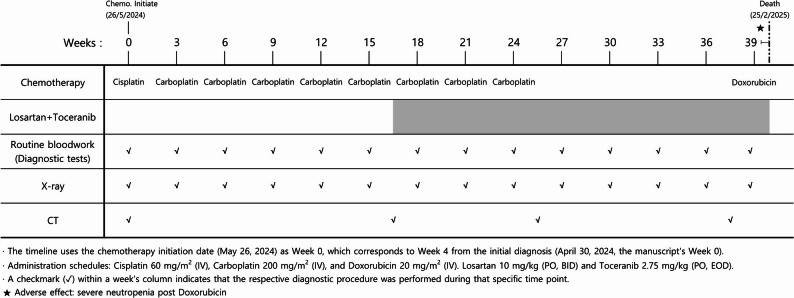
A detailed clinical timeline showing the chronological progression of the multimodality therapeutic protocol, specific monitoring time points, and key outcomes. This figure illustrates the chronological progression of the therapeutic regimen, encompassing the initial chemotherapy (cisplatin followed by carboplatin), the introduction of the triple-combination regimen (high-dose losartan, toceranib, and carboplatin), and the final administration of doxorubicin, which resulted in a severe adverse effect (marked by ★). It also details the schedule for routine monitoring, including blood work, X-rays, and CT scans

Adjuvant chemotherapy was initiated on May 26, 2024 (Week 4 from diagnosis) approximately two weeks after post-amputation (Fig. 2). The initial regimen consisted of one cycle of cisplatin administered intravenously (IV) at a dose of 60 mg/m². Due to concerns regarding potential nephrotoxicity, the chemotherapy protocol was subsequently changed to carboplatin (200 mg/m² IV every 21 days). During chemotherapy, serial complete blood counts (CBCs) and serum biochemical profile were performed periodically for two distinct purposes: first, to monitor for anticipated chemotherapy-induced neutropenia (nadir checks), typically 7 to 10 days post-chemotherapy; and second, every three weeks immediately prior to the next scheduled chemotherapy administration, primarily for overall toxicity assessment and data reporting consistency (as presented in Table 1). The routine bloodwork row in Fig. 2 reflects the three-week interval used for reporting. Thoracic radiographs were obtained every three weeks to routinely assess pulmonary metastatic lesions, and a CT scan was performed after the completion of the six cycles of cisplatin and carboplatin adjuvant chemotherapy, confirming progressive pulmonary metastasis on September 24, 2024 (21 weeks from diagnosis, following the completion of week 18 chemotherapy).

**Table 1:** Changes in hematological and biochemical parameters during treatment

Variables (RI)	Day 0	Week 3	Week 6	Week 9	Week 12	Week 15	Week 18	Week 21	Week 24	Week 27	Week 30	Week 33	Week 36	Week 39
RBC (5.65–8.87 10^12^/L)	N/A	8.09	8.21	9	9.37	8.87	N/A	8.61	8	7.05	7.64	8.23	8.51	6.88
Neutrophil (2.95–11.64 10^9^/L)	N/A	3.3	3.1	3.4	3.8	4.37	N/A	3.77	5.21	0.25	1.91	3.23	2.51	0.47
Platelet (148–484 10^9^/L)	N/A	189	302	338	256	242	N/A	125	258	120	236	228	246	236
BUN (9.2–29.2 mg/dL)	23	29	29	N/A	32.1	27	N/A	24.2	24.3	24.7	23.3	28.3	24.6	N/A
CREA (0.4–1.4 mg/dL)	1.7	2.3	2.5	N/A	1.57	1.82	N/A	1.32	1.5	1.29	1.59	1.3	1.42	N/A
ALP (47–254 U/L)	N/A	N/A	N/A	N/A	N/A	N/A	N/A	38	35	33	23	63	128	N/A
ALT (17–78 U/L)	N/A	N/A	N/A	N/A	N/A	N/A	N/A	31	26	32	28	N/A	43	N/A
AST (17–44 U/L)	N/A	N/A	N/A	N/A	N/A	N/A	N/A	46	36	N/A	N/A	N/A	N/A	N/A
cCRP (0–10.00 mg/L)	N/A	5.94	N/A	4.9	N/A	N/A	N/A	37.13	18.07	6.58	6.43	17.62	23.67	61.39
D-dimer (0-250 ng/mL)	109.1	N/A	N/A	76.01	N/A	N/A	N/A	334.5	239.2	81.58	82.17	492.9	433.1	343.3

N/A indicates no test result

*Abbreviations*: *RI* Reference interval, *RBC* Red blood cell, *BUN* Blood urea nitrogen, *CREA* Creatinine, *ALP* Alkaline phosphatase, *ALT* Alanine aminotransferase, *AST* Aspartate aminotransferase, *cCRP* canine C-reactive protein, *D-dimer* Degradation fragment

**Fig. 3:**
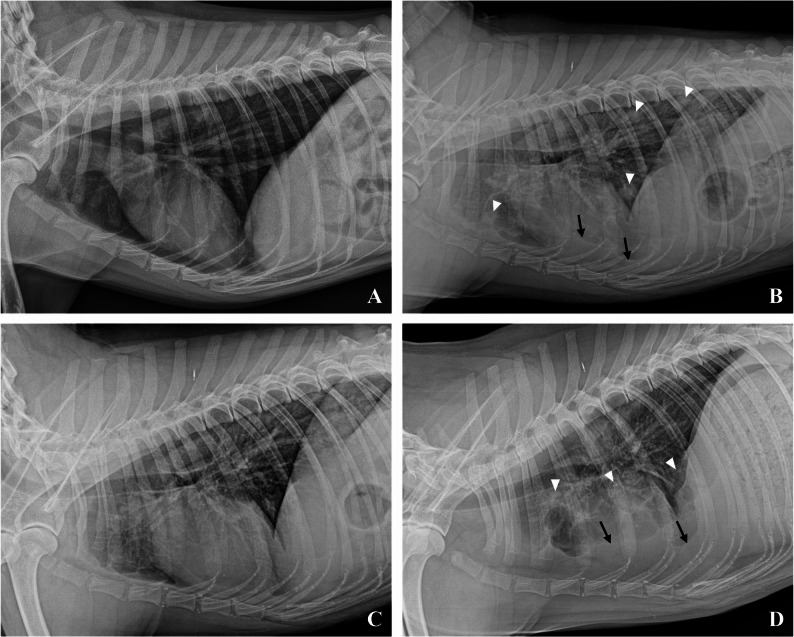
Temporal changes in pulmonary nodules and pleural effusion on left lateral thoracic radiographs from a dog with osteosarcoma and pulmonary metastasis before and after the triple-combination regimen (high-dose losartan, toceranib, and carboplatin) administration. (**A**) At baseline, no radiographically detectable pulmonary nodules or pleural effusion are identified. (**B**) Four months later, the radiograph shows multiple pulmonary nodules (soft tissue opacity, well-defined margins, white arrowheads) throughout the lung fields. Pleural effusion (black arrows) is also present. (**C**) Two months after beginning the triple-combination regimen, pulmonary nodules are no longer radiographically visible, and the previously noted pleural effusion has resolved. (**D**) At follow-up, after clinical deterioration, recurrent pleural effusion (black arrows) and multiple pulmonary nodules (white arrowheads) are again identified; some nodules are less conspicuous and partially obscured by summation with intrathoracic structures consistent with disease progression

During the first four months of this regimen, routine monitoring revealed stable hematological parameters and unremarkable serum biochemical results. Furthermore, canine C-reactive protein (cCRP) and D-dimer levels remained within the normal range, reflecting the patient’s clinical stability during the initial phase of adjuvant therapy. The initial thoracic radiographs also showed no detectable pulmonary nodules (Fig. 3A).

**Fig. 4:**
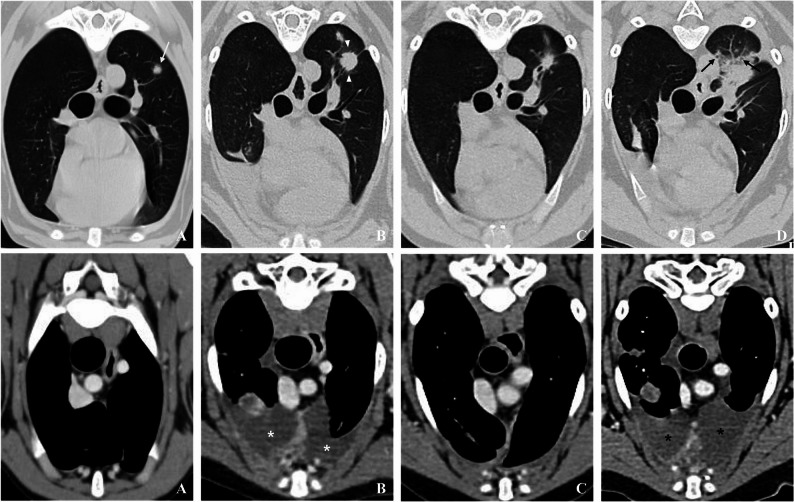
Sequential transverse CT images of the thorax demonstrating the progression of pulmonary metastasis and changes in pleural effusion in a canine osteosarcoma. The upper panels illustrate the temporal evolution of pulmonary metastatic nodules, while the lower panels depict changes in pleural effusion volume over time. All images were acquired using 1.0-mm slice thickness. Upper panels (Pulmonary metastatic nodules): These images are displayed with a lung window (window level − 400; window width + 1,500). (**A**) Baseline CT scan obtained during initial presurgical staging (corresponding to the time of diagnosis, week 0). The image shows a solitary soft tissue–attenuating nodule in the left caudal lung lobe (white arrow). This finding established the presence of pre-existing pulmonary metastasis. (**B**) At the 4-month follow-up, multiple nodules of increasing size and number are seen. Some lesions had a peripheral ground-glass halo (white arrowheads). (**C**) Two months after the initiation of the triple-combination regimen, a partial regression response is evident, as indicated by a significantly decreased nodule size and attenuation. (**D**) Re-evaluation following clinical deterioration highlights extensive disease progression, with numerous coalescing nodules exhibiting ill-defined, spiculated margins, and internal air bronchograms (black arrows). Lower panels (pleural effusion volume): These images are displayed using a soft-tissue window (window level + 40; window width + 400). (**A**) Baseline CT showing no pleural effusion. (**B**) At the 4-month recheck, bilateral pleural fluid accumulation is evident (white asterisks). (**C**) Two months after the initiation of the triple-combination regimen, a substantial reduction in pleural fluid volume is observed. (**D**) At reevaluation following clinical deterioration, the recurrence of pleural effusion is noted (black asterisks)

A follow-up radiograph (Fig. 3B) taken four months after the initial diagnosis showed progression of the pulmonary metastasis, characterized by the appearance of multiple pulmonary nodules and an accompanying pleural effusion. This was corroborated by sequential transverse CT images (Fig. 4, Upper Panel B; Lower Panel B), which revealed an increase in the size and number of nodules along with bilateral pleural fluid. No distant skeletal or regional lymph node metastases were observed.

In response to disease progression, an alternative therapeutic strategy (Losartan/Toceranib/Carboplatin) was initiated on September 24, 2024 (21 weeks from diagnosis). A combination of high-dose losartan (10 mg/kg PO BID) and toceranib (2.75 mg/kg PO EOD) was initiated concurrently with the existing carboplatin cycles. Normal blood pressure was confirmed before the combination therapy was commenced. Throughout the combined treatment, CBCs and vital biochemical markers (renal and hepatic parameters, CRP levels, and other relevant markers) were carefully monitored. These markers showed stable trends with no statistically or clinically significant long-term fluctuations. This stability confirmed that no dose reduction or schedule delay was required for the Losartan/Toceranib and Carboplatin combined regimen.

Comprehensive CT re-evaluation revealed a significant positive response on November 25, 2024 (30 weeks from diagnosis). Thoracic radiographs (Fig. 3C) showed that the pulmonary nodules were no longer visible and the pleural effusion resolved. This impressive regression was further corroborated by sequential transverse CT images (Fig. 4, Upper Panel C; Lower Panel C), which demonstrated a partial response with decreased nodule size and attenuation and a substantial reduction in pleural fluid volume. During this period, D-dimer and cCRP levels showed a notable decrease, returning to a near-normal range, corresponding to the observed clinical improvement.

Encouraged by these positive results, the patient was switched to losartan and toceranib combination therapy after the completion of carboplatin cycles. However, on February 7, 2025 (40 weeks from diagnosis), during the 3rd post-operative CT examination, the patient’s clinical condition deteriorated. Clinically, a noticeable decline in vitality and appetite was observed, accompanied by harsh breathing sounds and reduced exercise tolerance. The CT tomography confirmed disease progression, showing an increase in both the size and number of pulmonary metastatic nodules, with the largest increase being approximately 30%. Recurrent pleural effusion and multiple pulmonary nodules were also observed on subsequent radiographs (Fig. 3D) and computed tomography (CT) scans (Fig. 4, Upper Panel D; Lower Panel D), consistent with extensive disease. At this time, the D-dimer and cCRP levels showed a marked increase, reflecting renewed disease progression.

Doxorubicin was administered as a third-line chemotherapeutic agent on February 19, 2025 (42 weeks from diagnosis). The patient’s condition rapidly deteriorated following treatment. Significant adverse effects, including severe vomiting and a life-threatening grade 4 neutropenia (0.47 × 10^9^/L; based on VCOG-CTCAE v2 criteria), were noted. The patient ultimately died six days later, on February 25, 2025 (43 weeks from diagnosis).

In summary, the patient progressed after approximately five months (21 weeks) on first-line chemotherapy, achieved a profound partial response for approximately nine weeks (2 months) on the triple-combination regimen (Losartan, Toceranib, and Carboplatin), progressed again after approximately 2.5 months (10 weeks) on oral combination therapy, and ultimately succumbed to the disease approximately 10 months (43 weeks) after the initial diagnosis.

## Discussion

Canine OSA remains a formidable challenge in veterinary oncology because of its aggressive nature and the high metastatic risk, particularly in the lungs [1–3]. Despite established standard-of-care treatments, the prognosis for affected patients remains poor [1–4]. The development of innovative therapeutic strategies to improve patient outcomes is urgently needed.

This case highlighted the limitations of conventional chemotherapy in the management of advanced metastatic OSA. After 18 weeks (four months) of adjuvant carboplatin therapy, the patient’s pulmonary metastasis progressed, underscoring the need for an adapted therapeutic approach. This finding is consistent with the variable and often limited efficacy of traditional cytotoxic agents against metastatic diseases. It is also important to note that throughout this period, while the hematological parameters remained relatively stable, the patient experienced periods of transient myelosuppression following carboplatin cycles. However, no sustained long-term trends were observed in renal or hepatic function. Alternative strategies for managing metastatic canine OSA, such as metronomic chemotherapy, aim primarily for disease stabilization and quality of life enhancement rather than cure, with recent studies examining protocols combining oral cyclophosphamide with agents like toceranib or novel anti-neoplastics [10, 11].

In response to disease progression, we initiated the triple-combination regimen of high-dose losartan(ARB), toceranib, and carboplatin, guided by Regan et al. [7]. This strategic decision proved highly impactful. A comprehensive CT re-evaluation nine weeks (30 weeks from diagnosis) into this regimen revealed a significant positive therapeutic response. The pulmonary nodules became radiographically invisible, and the previously observed pleural effusion was completely resolved (Figs. 3C and 4C). This substantial improvement strongly suggests a synergistic anti-cancer effect of the losartan and toceranib combination, likely in conjunction with the concurrent carboplatin cycles, against aggressive pulmonary metastasis, a level of response that is unlikely to be achieved with either agent alone. The synergy is hypothesized to stem from losartan’s capacity to remodel the tumor microenvironment (TME), thereby enhancing the anticancer efficacy of toceranib [7]. This remarkable clinical response, mirrored by a notable decrease in D-dimer and cCRP levels, not only highlights the potential of this novel combination therapy in veterinary medicine but also reinforces the value of canine osteosarcoma as a model for advancing therapeutic strategies in human oncology [9].

This positive outcome further emphasizes the critical role of TME in OSA progression and therapeutic response, which is consistent with recent findings [12]. Losartan, an ARB, modulates the TME by reducing desmoplasia and immune cell infiltration [13]. Given the complex immune landscape of OSA, in which the TME significantly influences tumor behavior, the ability of losartan to alter this permissive environment likely amplifies the effectiveness of toceranib. Toceranib, a multi-targeted tyrosine kinase inhibitor [8], not only directly targets tumor cells and angiogenesis, but may also operate more efficiently within a TME rendered less immunosuppressive or fibrotic by losartan, indicating a potential synergistic interaction beyond its individual mechanisms [7, 12].

This observation aligns with the growing translational interest in human oncology, where canine cancer research is increasingly driving drug repurposing efforts for human osteosarcoma [9]. Specifically, the strategy of combining traditional cytotoxic chemotherapy with agents that modulate the tumor microenvironment, such as the use of ARBs as co-adjuvant therapy in cancer, is under investigation to enhance overall treatment efficacy in refractory osteosarcoma [3, 13]. However, despite the initial success achieved with the triple-combination regimen, the patient’s condition ultimately deteriorated, and disease progression was confirmed during the re-evaluation CT examination on February 7, 2025 (40 weeks from diagnosis) (Figs. 3D and 4D). This underscores the inherent virulence of canine OSA and the challenges in achieving durable long-term remission, even with novel and seemingly effective combinations. Unfortunately, a subsequent attempt with doxorubicin as a third-line agent led to severe adverse effects and rapid decline, culminating in the patient’s death on February 25, 2025 (43 weeks from diagnosis). This final decline included severe neutropenia (0.47 × 10^9^/L) and a peak cCRP level (61.39 mg/L) following doxorubicin administration.

Crucially, the overall survival of 43 weeks (301 days) achieved by this patient represents a significant clinical benefit, substantially surpassing the poor prognosis for dogs with macroscopic pulmonary metastasis (Stage III disease), which is generally reported to range from 2 to 3 months (approximately 59 to 76 days) despite treatment [5, 6]. The total duration of disease control from the initiation of the triple-combination regimen until final progression was 19 weeks. This duration is highly favorable, being comparable to the median progression-free survival (PFS) of 21 weeks reported in the Regan et al. study for the losartan/toceranib combination. Furthermore, the patient maintained progression-free status for 10 weeks even after transitioning to oral combination therapy, a period that exceeds the median PFS of approximately 8 weeks reported for single-agent toceranib in comparative studies. This robust response highlights the potential long-lasting tumor microenvironment modulation effect derived from the initial, intense triple combination therapy.

Although our case provides notable clinical observations, it is essential to acknowledge several inherent limitations. First, as a single-case report, the findings have limited generalizability to the broader canine OSA population, and the absence of a control group precludes a direct comparison with the natural disease course. Second, although the dosages were based on established protocols for individual or dual therapies, the concurrent administration of carboplatin makes it difficult to ascertain the precise contribution of each agent. Specifically, there is currently a lack of large-scale data regarding the optimal dosing, safety, and potential pharmacokinetic interactions when these three drugs are combined. It remains to be determined whether the positive outcome resulted from the losartan/toceranib combination alone or a synergistic interaction with ongoing chemotherapy. Therefore, future prospective trials are warranted to fully delineate optimal therapeutic strategies and to standardize the triple-combination regimen for refractory canine osteosarcoma.

## Conclusion

In conclusion, this case report presents a clinical observation regarding the potential therapeutic value of the triple-combination regimen of high-dose losartan, toceranib, and carboplatin for progressive pulmonary metastasis in canine OSA, where we observed a significant response in which standard single-agent chemotherapy had failed. The initial profound response, likely augmented by favorable TME modulation, supports the exploration of drug repurposing for anticancer properties in combination with targeted therapies. Although this single case demonstrated a promising initial effect, the ultimate disease progression highlights the ongoing need for continuous treatment adaptation and underscores the limitations of achieving sustained control in such aggressive cancers. Larger prospective studies are needed to fully evaluate the long-term efficacy, optimal dosing, and generalizability of this combination therapy, thereby contributing to the broader development of innovative multimodal approaches that account for the intricate interplay within the tumor microenvironment to effectively combat canine osteosarcoma.

## Data Availability

Data sharing is not applicable to this article because no new datasets were generated or analyzed during the current study owing to patient privacy concerns.
